# Refining a Research Manuscript

**DOI:** 10.4103/0975-1483.66788

**Published:** 2010

**Authors:** Ambikanandan Misra

**Affiliations:** *Professor in Pharmacy (Pharmaceutics), Pharmacy Department, Faculty of Technology and Engineering, The Maharaja Sayajirao University of Baroda, Post Box No.: 51, Kalabhavan, Vadodara - 390 001, Gujarat, India. E-mail: misraan@hotmail.com*

A well-written research article is quintessential for the innovative work to be appreciated and receiving a good number of citations. But writing a good research article is not an art. With due care and understanding the focus requirements can help a researcher to present his work with high scientific impact. The seeds of a research article are in the primary research or discovery by a researcher, which once communicated and peer reviewed takes the form of a publication and triggers further scientific pursuits in future.

Before beginning to draft a research manuscript, it is important to decide upon the journal a researcher wants to publish his work in. The draft can be then prepared according to its format requirements. But a standard flow of information in any article is as follows: abstract followed by introduction, details of materials and methods and then results leading to discussion and conclusion and ending up with acknowledgments and references. A research report is an objectively written piece where no personal anecdotes are included. The research article should not be written in first person and it should be attempted that there are least number of grammatical mistakes or mis-spellings in the article. The choice of words may also affect the impact or curiosity generated from the article, fancy words are better avoided and words reflecting knowledge like define, describe, identify, know, label, list, match, outline, recall, recognize, state, locate, find, show, use, illustrate, construct, examine, explain, interpret, outline, discuss, distinguish, predict, restate, translate, compare are appropriate for use.

## TITLE OF THE ARTICLE

The title of the article describes the focus of the work reported in the manuscript and helps future researchers to go through the article. It is very important to select an appropriate title, which should be self explanatory. An attractive title invites attention of the readers towards the manuscript and the content of the manuscript should be enough to keep the interest of the reader. Too catchy title is not always beneficial in case the contents of the manuscript are weak because more readers also mean more negative impact.

## ABSTRACT OR SUMMARY

The article begins with the abstract, which is summary of the research work. It is a concise and focused representation of research work indicating the aim of the study, the methods used, results and major findings as a conclusion from the studies. It ends with the implications for future. The abstract gives a first-hand impression of the impending article to the reader and needs to be written precisely and with clarity. The researchers should refrain from writing extended background or methodology. Few keywords may be given at the end of abstract representing the field of significance of the article and will help future researchers in searching for appropriate references. Abstract is a must for every article and missing information or too much description are the common errors. The abstract must follow – introduction – purpose – methodology – results – conclusion – future implication format.

## INTRODUCTION

Introduction describes the motivation for the studies. The article starts with the introduction to the reader of the shortcomings or lacunae in the current state of art in the identified research field. It leads to the objective of the proposed work and the hypothesis on which the work is based. Initially, the researcher presents the background in the field and the gravity of the problem and any earlier attempts by other researchers to build upon his hypothesis. The format that should be followed is introduction to the broad topic, the problem and background and advance to a narrow topic to state his hypothesis. Previous studies should be discussed to make the reader understand the current status in the field but should not be too extensive. It should be relevant and just enough. Researchers should describe how their studies or innovation will fill the technological gap. They should explain important theoretical concepts implied in the study. An introduction is around 400 to 500 words long. Thus, the introduction is a brief account of previous research, clear statement of the problem, proposal of a hypothesis and moves on to state the significance of the studies conducted. Introduction should not give a confused outlook, presenting too much or too less information or unclear statements.

## MATERIALS AND METHODS

Usually, introduction is followed by the materials and methods section. It should provide the technical grade or specifications of all the materials used along with their sources. It may be noted that materials are available in different grades of the same chemical compound or substance and can influence the outcome of the studies. Many a times, special equipments used and their model and manufacturer’s detail may be incorporated. In methods section, a thorough description of the methodologies used for carrying out the proposed work is provided. In case, the method is been used from standard books or references, then the method may be written in short with appropriate references. This allows readers to repeat the experiments as stated in the manuscript or through literature citations. Methods should be written with sufficient details with clarity for the reference of future researchers in the same field. To summarize, this section should detail the materials used, the experimental methods, equipments and instruments used with their model numbers and if any software that has been used. It should document the permissions obtained for carrying out any animal or clinical studies from appropriate ethical committees and Drug Controller General of India, registration with Indian Council of Medical Research, use of the subjects, their origin and handling, the techniques used for the sample preparation, method of data collection, use of any positive or negative controls in the study and statistics applied to interpret the data. The section should be balanced and focused, avoiding background information from introduction, results or reporting of errors.

## RESULTS

Results section follows the methodology section and reports the findings from the experiments, tests, models or theories applied during the research work. The significant results should be documented in text and additionally, tables and figures should be used to present the result data. However, it is very essential to refrain from drawing conclusions or interpretations, if a distinct discussion and conclusion section is requisite according to the instructions to author. It is only to present and illustrate findings of objectives of the studies. There should be sufficient and appropriate details. Results should be presented very intelligently avoiding raw data and calculations and should summarize the results in tables and figures for better clarity and understanding at a glance. The text should highlight significant findings, trends and figures and tables may be briefly explained. The results should be explained with the details of number of repeat trials / experiments and the statistics applied on them. A simplistic and appropriate statistical model should be used. Researcher must take extreme care to avoid repetition until and unless needed for clarity. Results that are only crucial to studies should be written in text to prove proposed hypothesis. Results may be described by providing a context-like, stating the problem to which it is addressed.

## DISCUSSION AND CONCLUSION

The discussion and conclusion section should interpret in-depth the results from the studies carried out. The researcher may begin by brief description of the problem, proposed studies that are going to be addressed and restatement of the hypothesis. The significant results should be discussed in light of prior art in the field with the references, and can be used to justify the hypothesis. The researcher may agree or disagree with the previous studies. Observed results may or may not have any similarity or connectivity to any previous studies or contrary to expectations. In such circumstances, researchers should try to justify the results with logical and scientific explanation. However, overgeneralization or superficial interpretation may be avoided in absence of authentic data or literature backing. It should also be explained that whether these studies were effective to test the hypothesis and any additional studies if required. The discussion part should also state the possibility of errors / anomalies in the results explaining its source that is, the limitations of studies. A clear-cut realistic conclusion based on proposed hypothesis should be made. It should move on to relate researchers’ results to the impact it will have on the understanding and advancement of the subject under study. Conclusions can include the possible applications of the research and researchers’ perception of the future direction to the work to be carried out by other researchers. No new results should crop up in discussion and conclusion section and a researcher must avoid broad statements.

